# Classification of Paediatric Inflammatory Bowel Disease using Machine Learning

**DOI:** 10.1038/s41598-017-02606-2

**Published:** 2017-05-25

**Authors:** E. Mossotto, J. J. Ashton, T. Coelho, R. M. Beattie, B. D. MacArthur, S. Ennis

**Affiliations:** 1 Human Genetics and Genomic Medicine, University of Southampton, Southampton, UK; 2 Institute for Life Sciences, University of Southampton, Southampton, UK; 3 Department of Paediatric Gastroenterology, Southampton Children’s Hospital, Southampton, UK

## Abstract

Paediatric inflammatory bowel disease (PIBD), comprising Crohn’s disease (CD), ulcerative colitis (UC) and inflammatory bowel disease unclassified (IBDU) is a complex and multifactorial condition with increasing incidence. An accurate diagnosis of PIBD is necessary for a prompt and effective treatment. This study utilises machine learning (ML) to classify disease using endoscopic and histological data for 287 children diagnosed with PIBD. Data were used to develop, train, test and validate a ML model to classify disease subtype. Unsupervised models revealed overlap of CD/UC with broad clustering but no clear subtype delineation, whereas hierarchical clustering identified four novel subgroups characterised by differing colonic involvement. Three supervised ML models were developed utilising endoscopic data only, histological only and combined endoscopic/histological data yielding classification accuracy of 71.0%, 76.9% and 82.7% respectively. The optimal combined model was tested on a statistically independent cohort of 48 PIBD patients from the same clinic, accurately classifying 83.3% of patients. This study employs mathematical modelling of endoscopic and histological data to aid diagnostic accuracy. While unsupervised modelling categorises patients into four subgroups, supervised approaches confirm the need of both endoscopic and histological evidence for an accurate diagnosis. Overall, this paper provides a blueprint for ML use with clinical data.

## Introduction

Paediatric inflammatory bowel disease (PIBD), comprising Crohn’s disease (CD), ulcerative colitis (UC) and inflammatory bowel disease unclassified (IBDU) are a group of autoimmune inflammatory conditions affecting children, the incidence of which is increasing^1, 2^. The major feature of inflammatory bowel disease is chronic inflammation of the gastrointestinal (GI) tract. Symptoms of PIBD include diarrhoea, abdominal pain, blood in the stool and weight loss^3^. Although both Crohn’s disease and ulcerative colitis are considered to fall within the same disease group, there are often differences in disease location within the bowel, observable through endoscopic and histological assessment. Endoscopic investigation of disease is macroscopic and typically determines initial treatment and provisional diagnosis, however the endoscopic assessment of the gastrointestinal system is not always sufficient for diagnosis and histological (microscopic) examination of biopsies from the upper and lower GI tracts is vital to determine disease extent and confirm diagnosis. Typically, Crohn’s disease is characterised by a non-continuous inflammation of the entire gastrointestinal system, while the inflammation pattern of ulcerative colitis is continuous and restricted to the colon and rectum. There is a well-established discordance between endoscopic (macroscopic) and histological (microscopic) disease extent^4–6^. Mucosal healing (histological) is frequently cited as a ‘true’ measure of remission. Despite this, the major clinical classification tool for PIBD, the Paris classification, is based exclusively on endoscopic and radiological disease extent^7–9^. Previous data has indicated histological disease extent to be significantly greater than endoscopic disease extent, at both diagnosis and follow-up^4, 5^. This raises the possibility of a modification of classification to account for histological disease as an additional measure of disease extent. However, the current endoscopic Paris classification remains a validated tool to guide treatment^6, 10^.

Diagnosis of PIBD is challenging, the aetiology is not fully understood and deciding on management and prognostication is complex. The accuracy of diagnosis in PIBD is key to prompt and effective treatment^11^. The treatment for PIBD is highly dependent on disease location and disease extent, as well as accurately classifying as CD, UC and IBDU. Surgical intervention may be necessary for pancolitis in UC but would not provide a cure for pancolitis in CD. Additional decisions about escalation of therapy, including use of monoclonals, rely on precise understanding of an individual patient’s disease. The use of these therapies is not without drawbacks and accurate diagnosis is vital to achieve remission without putting the patient at risk of harm.

Uncertainty in the classification or the severity/extent of disease can lead to delays or inappropriate treatment^12^. Tools to assist clinicians in making a more accurate diagnosis are attractive and may assist in the better categorisation of disease into a number of specific phenotypes with implications for how best to treat. Plevy *et al*. previously developed a multi-component machine learning model (including serological and genetic markers) in adult IBD to assist with diagnosis achieving good CD/UC discrimination^13^. However, these markers are expensive, time consuming to generate and not routinely available in most hospitals; to date there are no mathematical models based solely on simple clinical data such disease location to assist with diagnosis and classification.

Machine learning is a contemporary branch of statistics particularly well suited for analysis of complex data. Machine learning algorithms aim to find patterns within data and use them to make predictions and classifications or infer new knowledge^14^. These methods are broadly grouped in two categories: (1) unsupervised machine learning algorithms do not need *a priori* knowledge of classes, instead they aim to infer classes on the basis of presenting features; (2) supervised algorithms are better suited to solve classification problems where the class of each sample/patient is known *a priori* – these samples are then used to train a model to classify subsequent samples of *unknown* class. This study utilises unsupervised models to examine the evidence for clearly distinguishable strata identifiable through endoscopic and histopathological data and examines the properties of any inferred groups. The study then applies a supervised support vector machine (SVM) and patient samples with established diagnoses of either CD or UC to construct a classification model. The resultant model is tested for accuracy and implemented on an unseen validation cohort. Such methodology has been used successfully in medicine and biology for cancer subtype classification, novel drug discovery and genomics^15–19^. Here we use paediatric patient endoscopic and histological data to assess the utility of such approaches for the diagnosis and management of this complex disease.

## Materials and Methods

Patients were recruited from the Genetics of Paediatric Inflammatory Bowel Disease study at Southampton Children’s Hospital. Data were collected from prospectively entered electronic clinical records using a standardised proforma^5^. Fully anonymised patient data were obtained from endoscopy and histology at initial diagnosis, all patients were diagnosed in line with Porto criteria^20^. Disease type was confirmed by two investigators (RMB, JJA). The dataset comprised manually collected data from 287 patients, 178 with Crohn’s disease, 80 with ulcerative colitis and 29 with inflammatory bowel disease unclassified (Supplementary dataset 1). The ratio of CD to UC is typical of paediatric onset disease^2^.

Informed consent was obtained for all participants. The study has full ethical approval from Southampton & South West Hampshire Research Ethics Committee (09/H0504/125). All methods were performed in accordance with the relevant guidelines and regulations.

Ten gastrointestinal (GI) locations were investigated for the presence of macroscopic and microscopic abnormalities: mouth, oesophagus, stomach, duodenum, ileum, ascending colon, transverse colon, descending colon, rectum and perianal. Clinical observations were converted into numerical variables [−1, 0, +1] depending on tissue abnormalities. At each location, abnormal tissues observations were coded as +1 and normal were coded as −1. Null values (0) were assigned for missing data such as in the case of restriction at endoscopy. Mouth and perianal locations are not typically biopsied for histology, therefore these feature were excluded in the unsupervised approach and automatically excluded in the supervised approach.

### Unsupervised machine learning

In order to observe whether clinical features can induce the formation of two clusters representing CD and UC, data were modelled using principal component analysis (PCA) and multidimensional scaling (MDS) algorithms as unsupervised machine learning approaches. In unsupervised machine learning the diagnosis of CD, UC or IBDU is hidden from the model, leaving the algorithm to impose the most relevant strata. Both PCA and MDS are dimensionality reduction algorithms that convert a high dimensional space (here each dimension corresponds to a measured traits), to a lower dimensional space (usually 2D or 3D). The main difference between PCA and MDS is the search space of those two algorithms. While PCA investigates linear feature associations, MDS can also uncover non-linear associations. However, if the associations between the features are essentially linear then multidimensional scaling will provide a similar representation to that of PCA.

To better visualise the relationship between patients and traits, hierarchical clustering with Hamming distance^21^ and average linkage^22^ was performed.

Groups identified by hierarchical clustering were assessed with respect to: age of onset and C-reactive protein levels at diagnosis, using ANOVA; disease subtype, gender, family history and personal history of autoimmune disease using χ^2^. Statistical analyses were performed applying Python SciPy package^23^.

### Supervised machine learning

In order to discriminate CD and UC patients, a model was assembled utilising different techniques of supervised machine learning. We applied a supervised machine learning model where the diagnosis of CD and UC was seen by the model.

In order to isolate the key histological and endoscopic features that determined diagnostic subgrouping, we tested a range of classification strategies including ensemble learners (Boosted and Bagged Trees), linear discriminant analysis and support vector machines (SVMs) with a variety of different kernels^14, 24^.

**Figure 1:**
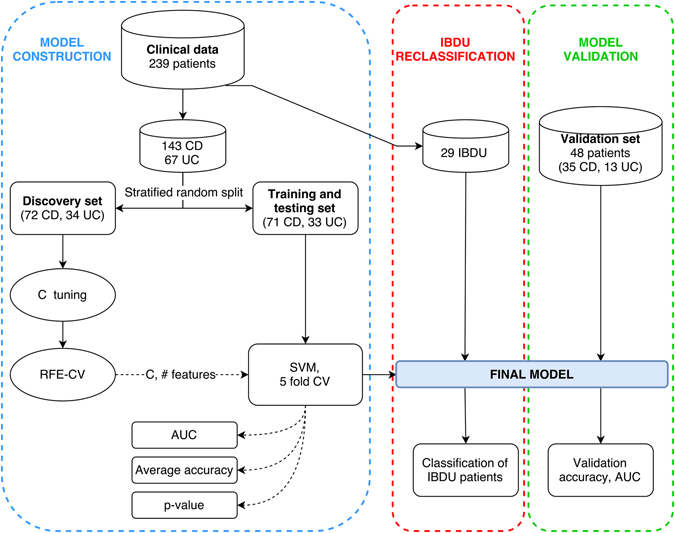
Model and data processing. Schematic representation of the model construction (blue section), validation (green section) and IBDU reclassification (red section) phases. Solid arrows represent data stream while dashed arrows represent parameters or metrics stream. The discovery set was used to identify the optimal penalty parameter (C) and number of features using the recursive feature elimination with cross validation algorithm (RFE-CV). These two elements were then passed to the training and testing set which was then modelled using a support vector machine (SVM). Three metrics were collected: area under the ROC curve (AUC); accuracy over the 5 folds and; a permutation-generated p-value.

Data were split in order to construct and then validate the model, 210 patients (n_CD_ = 143; n_UC_ = 67) patients were included in the model construction step. Forty-eight patients (n_CD_ = 35; n_UC_ = 13) were set aside to validate the model on unseen data. Data from IBDU patients (n = 29) were used only for a final reclassification. Figure 1 is a schematic representation of the model and shows the usage of the different subsets.

To create a model which is applicable to unseen data, the 210 CD and UC samples were randomly split in two subsets preserving the original disease subtype ratio. The first data subset was used for searching the best parameters for the CD *versus* UC classification (discovery set). The second data subset was used for training and testing the model according to the parameters determined during the discovery phase. After assessing the performance of the final model, data from IBDU patients were passed to the model in order to classify them as either CD or UC.

Construction of optimal model utilised a linear support vector machine, allowing for regression of weights for each feature and assessment of the relative importance of each variable. Additionally, linear SVMs require estimation of a single penalty parameter (C) that allows for misclassification within the training set. In an attempt to improve model performance when optimizing the classifier we allowed the search space for C values to range from 1 × 10^−3^ to 1 × 10^2^. Large values of C are less prone to misclassify data points, but perform suboptimally when classifying outliers in unseen data. Small C values generate models that are more robust to outliers by allowing more misclassified data points at the expense of the training accuracy.

Machine learning approaches are weakened by the inclusion of features that are not relevant to the classification problem (confounding factors or ‘noise’) and reduce model performance. In order to minimise noise from non-informative features, we applied a recursive feature elimination algorithm combined with a 5-fold cross validation scheme (RFE-CV) selecting pertinent features as described by Guyon *et al*.^25^. Including a 5-fold cross validation avoids overfitting the model to the discovery set by selecting parameters and features that are specific to this set but do not generalize well, and therefore perform poorly on the test subset. The selection of the best feature subset and optimal C were chosen to maximise the classification accuracy over the discovery set.

Following the identification of the optimal C and set of features, we trained a new support vector machine and tested its efficiency (Fig. 1). With a 5-fold cross-validation scheme the algorithm repeatedly fitted and tested data from the training/testing set, providing the average accuracy in the CD *vs*. UC classification. The area under the receiver operating characteristic curve (AUC) was used to assess model efficiency. Statistical significance of the observed accuracy was determined through permutation testing of 1,000,000 randomly generated models in which sample labels were shuffled. The p-value was then determined by calculating the frequency at which the observed accuracy was replicated by the random models. Finally, the overall performance of the model was verified by classifying unlabelled data from the validation dataset of 48 patients.

Once the model had been fully trained and validated, it was used to classify IBDU patients and posterior probabilities for membership to both the UC and CD classes were obtained. These probabilities depend on the distance between an observation and the decision function that SVM uses in order to discriminate between the two groups. The uncertainty in the classification of an individual increases as its profile is closer to the decision boundary (which is defined by the SVM decision function).

Data manipulation and modelling was performed using Matlab^24^ (R2016b), Python^26^ (2.7) and the Scikit-Learn^27^ (0.17.1) package.

## Results

Endoscopic and histological data were collected for 287 patients; 178 patients with Crohn’s disease, 80 with ulcerative colitis and 29 patients with inflammatory bowel disease unclassified. Machine learning was applied to 239 patients (CD = 143, UC = 97, IBDU = 29). Females account for 37% (107) of the individuals in the dataset. Average age of onset was 11.5 years (range 1.6 to 17.6 years). Twenty-six (9%) of patients were diagnosed below 6 years of age (very-early onset IBD). The remaining 48 patients (CD = 35, UC = 13, average age of onset 13.2 years) were used to validate the model.

### Unsupervised clustering shows the overlap of CD and UC phenotypes

**Figure 2:**
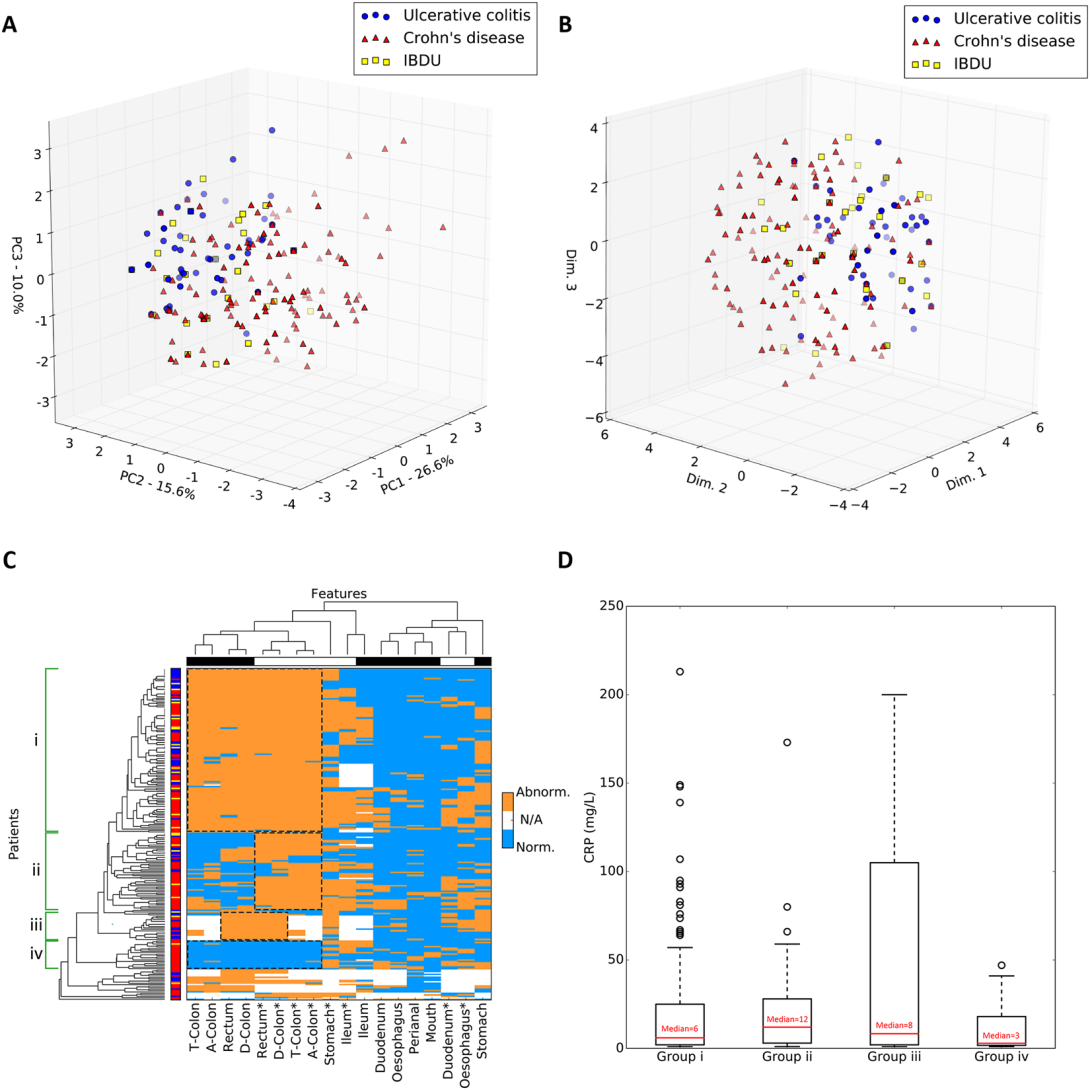
Dimensionality reduction approaches and hierarchical clustering of PIBD data. (**A**,**B**) Principal component analysis (**A**) and multidimensional scaling (**B**) of clinical data from 239 PIBD patients. The first three PCA components account for 52.2% of the total variance. Important note – UC/CD/IBDU diagnoses were used only to retrospectively colour data points and were not included in actual modelling. (**C**) Heatmap of endoscopic and histological tissue abnormalities in PIBD patients. Abnormal manifestations are shown in orange, normal in light blue and missing data in white. Asterisks indicate histology features. Ascending colon, transverse colon and descending colon labels were shortened to A-Colon, T-Colon and D-Colon respectively. Left hand side bar shows the referred diagnosis: CD in red, UC in blue, IBDU in yellow. Again, UC/CD/IBDU diagnoses were not used to model data but only to retrospectively colour each element. The top bar shows the type of investigation: histology in white, endoscopy in black. Identified colorectal groups are shown by dashed boxes and labelled from one (i) to four (iv). (**D**) Box and whisker plot depicting C-reactive protein (CRP) levels recorded at diagnosis across the four identified groups. Each box represents data from the first (bottom edge) and the third (top edge) quartile. Red bars and numbers are the median CRP level. Dashed whiskers show the lowest and highest CRP within each group. Black circles are outlier data points.

Endoscopic and histological data underwent principal component analysis with the first three components being representative of 52.2% of the total variance of data. According to both PCA and multidimensional scaling, there was no clear separation of Crohn’s disease and ulcerative colitis (Fig. 2A,B).

Despite the lack of distinct clusters, CD and UC individuals are differently distributed across the 3D space with regions predominantly populated by one or the other class. As anticipated, IBDU patients were distributed uniformly throughout the CD and UC data. The same clustering pattern was observed with MDS (Fig. 2B) strongly suggesting linear relationships between the measured features. The lack of clear clusters confirms the complexity in distinguishing CD and UC phenotypes from microscopic and macroscopic observations.

### Hierarchical clustering identifies four PIBD subtypes

In accordance with PCA and MDS analyses, hierarchical clustering did not stratify patients according to CD, UC and IBDU diagnosis (Fig. 2C). However, it did reveal the presence of distinct subgroups of patients, corresponding to complex patterns of abnormalities. As expected, most of the macroscopic and microscopic dysregulations were observed in the colorectal region. Considering only the colorectal region, it is possible to observe four distinct groups (Fig. 2C,i–iv). In the first group (i) patients exhibit tissue abnormalities identified by both endoscopy and histology. The second group (ii) shows colorectal abnormalities only after a microscopic investigation. Patients belonging to the third group (iii) present with inflammation of the rectum and the descending colon. Finally, the fourth group (iv) does not show any disruption of the colorectal region. Some patients are not placed within any of these four groups since they do not show any clear colorectal pattern. These patients have higher numbers of disease locations with null values (reflecting restriction at endoscopy).

The ileum exhibited an inconsistent pattern of disruption, acting as interface between mostly-abnormal and mostly-normal regions (left hand side *vs*. right hand side of Fig. 2C). Additionally, endoscopic or histological abnormalities in the upper GI tract are less frequent compared to lower GI tract abnormalities, this is equally applicable to all patients, regardless of their diagnosis (of CD or UC).

The four groups were analysed for any difference in their composition of patients with: a diagnosis of CD or UC; gender; positive or negative family history and clinical diagnosis of any other personal autoimmune disease. There was no significant difference between the groups with regard to any of these variables with the exception of diagnosis. Group iii (inflammation of the rectum and the descending colon) was significantly enriched for patients with ulcerative colitis patients (p = 0.046) and group iv (no colorectal involvement) was significantly enriched for patients with Crohn’s disease (p = 0.007). Groups i and ii were not significantly enriched either for CD or UC indicating presence of both disease types.

Regression analysis of the four groups identified a significant (p = 0.003) increase in CRP for patients in group iii compared to the other groups (Fig. 2D). There was no significant difference in age of diagnosis across groups.

### A combined model distinguishes Crohn’s disease from ulcerative colitis with the greatest accuracy

Model selection was based by testing a range of different algorithms and kernels. Table 1 reports classification accuracies obtained fitting and testing models on the whole dataset excluding IBDU patients and the validation cohort. Reported accuracies are only informative in terms of comparing different models and were not validated on external dataset. Linear discriminant and linear support vector machine outperformed other tested algorithms. Linear models performed better than Tree-based model and non-linear SVMs. Although 0.5% less accurate compared to a linear discriminant model, linear SVM represented the best choice in terms of adaptability and interpretation. Linear discriminant models assume data have the same covariance and a normal distribution, while SVMs does not have such requirements and is better suited for discriminative tasks^28^. Therefore, an SVM^14^ with a modified linear kernel was used as core classifier in our model.

**Table 1:** Preliminary assessment of linear and non-linear models. Linear support vector machine (SVM) was the selected model.

Method	Accuracy
Simple Tree (4 splits)	78.1%
Medium Tree (20 splits)	75.2%
Complex Tree (100 splits)	76.7%
Linear discriminant	81.0%
Linear SVM	80.5%
Quadratic SVM	78.1%
Cubic SVM	73.8%
Boosted Trees	74.8%
Bagged Trees	77.6%

In order to elucidate which observations are needed for optimal disease classification of patients, three supervised models were generated implementing endoscopic features, histological features and both endoscopic and histological features.

The combined model outperforms the other two models achieving the highest accuracy; the model correctly assigns the diagnosis of CD or UC to a patient in 82.7% of cases (Table 2). All metrics that assess model performance agree in the superior efficiency when using combined endoscopy and histology data. The combined model shows the highest accuracy, precision and F1-score; recall is close to that observed in the histological model. The endoscopy model performs well in terms of precision but is poorer in recall. Conversely, the histological model has the lowest precision but highest recall. This indicates that using endoscopy data the model is highly precise in identifying most of individuals from both classes (CD and UC). However, the endoscopy model is prone to produce more false negatives (recall) compared to the histology model. Both the accuracy and the F1 score, which combines precision and recall metrics, indicate that histology model is superior to the endoscopy model although having a lower precision. Moreover, the combined model selects all the features selected by the endoscopy and histology models plus two additional histological features (oesophagus and ascending colon). As expected, the ileum location appears to be consistently informative for the discrimination of CD and UC patients in every model, and in the histological model is sufficient to diagnose CD or UC in 76.9% of cases. Features with similar observations in both CD and UC patients are not informative for the classification while locations with a more variable manifestation of tissue damage were typically selected in the RFE-CV selection.

**Table 2:** Performance of the three optimised supervised models, asterisks indicate histological features.

Input	Accuracy % (AUC)	Precision	Recall	F1-score	(#) Features
Endoscopy	71.0% (0.78)	0.89	0.68	0.75	(5) Duodenum, Ileum, D-Colon, Rectum, Perianal
Histology	76.9% (0. 82)	0.81	0.86	0.83	(1) Ileum
Combined (E + H)	82.7% (0.87)	0.91	0.83	0.87	(8) Duodenum, Ileum, D-Colon, Rectum, Perianal, Oesophagus*, Ileum*, A-Colon*

All metrics represent the average over the 5-folds of the cross validation.

**Figure 3:**
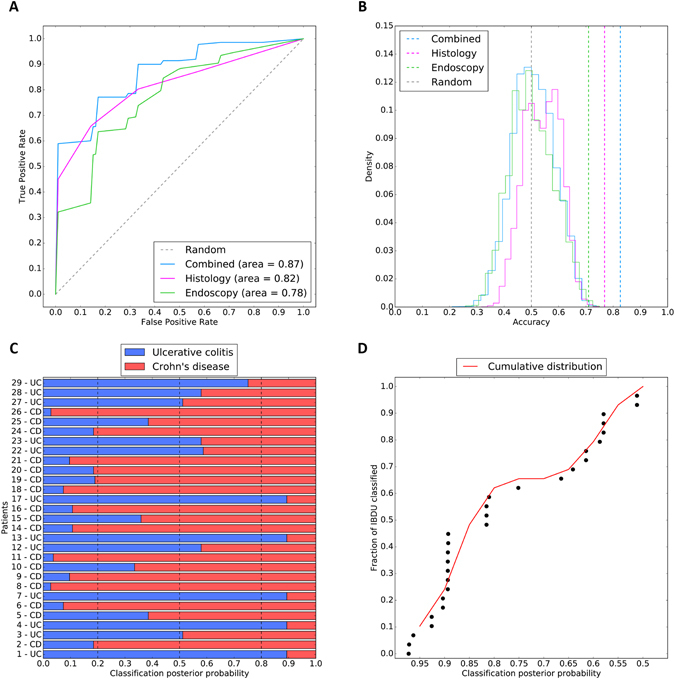
Supervised classification performance and metrics. (**A**) Receiver operating characteristic of the combined (light blue), histology (purple) and endoscopy (green) models. The grey dashed line represents the expected performance of a random model. (**B**) Permutation tests of models: dashed lines represent the observed accuracy of the combined (light blue), histology (purple) and endoscopy (green) models. The endoscopic, histological and combined models have a p-value of p = 3 × 10^−3^, p = 5 × 10^−6^ and p = 1 × 10^−6^ respectively. The grey dashed line represents the average expected performance of random model. Solid coloured lines show the distribution of random permutations for each model. (**C**) Classification of IBDU patients with the combined model in Crohn’s disease (red) or ulcerative colitis (blue) subtypes. The classification posterior probability indicates the confidence of the model in assigning UC or CD labels. (**D**) Cumulative confidence in IBDU reclassification represented as cumulative density function (red line) of posterior probabilities for 29 IBDU patients. Each dot represents an IBDU patient.

The greatest area under the curve (AUC) was observed in the combined model (0.87) followed by the histology (0.82) model and then the endoscopic model (0.78) (Fig. 3A). The endoscopic, the histological and the combined models showed a statistical significance of p = 3 × 10^−3^, p = 5 × 10^−6^ and p = 1 × 10^−6^ respectively (Fig. 3B).

For each training fold of the combined model, the observed accuracies (in decimals) were 0.86, 0.67, 0.95, 0.85 and 0.80 respectively. Overall, the mean accuracy was 0.83, the median 0.85, the standard deviation 0.09 and the standard error 0.05. Over the 1,000,000 permutations, none of the randomised models achieved an accuracy equal or greater than the observed (p-value = 1 × 10^−6^). These metrics indicate good overall performance and no overfitting of the model.

### Assessment of the combined model in an additional cohort

In order to further validate the combined histological and endoscopic model we applied it to classify 48 anonymised PIBD patients (validation set, Fig. 1). These data had not been used in the optimisation or training of the model. The model was accurate in classifying this additional cohort, correctly assigned the diagnosis of CD or UC in 83.3% of cases (Table 3). The performance metrics calculated on the validation set confirm the previous results in terms of accuracy and recall. However, precision, and consequently the F1-score, are lower when compared to the performance calculated over the test set. F1-score of the validation set is still higher than the histology and endoscopy only models.

**Table 3:** Performance of the trained combined model over the validation set.

Validation set	Accuracy %	Precision	Recall	F1-score	Support
UC	—	0.65	0.85	0.73	13
CD	—	0.94	0.83	0.88	35
Average/Total	83.3%	0.86	0.83	0.84	48

Since the validation set never took part in any phase of the model generation, and since the model was already trained and tested avoiding overfitting, the accuracy over the validation set did not required any additional shuffling.

### IBDU patients can be categorised by the combined model

The combined model was used to attempt to classify the 29 IBDU patients by assigning them to either a CD or UC subtype and computing the posterior probability of belonging to each class (Fig. 3C). It should be noted that the model was not trained to classify IBDU therefore patterns restricted to this class were not learnt by the algorithm. Instead the model aims to identify patterns learnt from UC and CD data in these previously unseen IBDU cases.

When applied to the 29 IBDU patients, 17 patients were assigned as Crohn’s disease and 12 as ulcerative colitis. In 17 of these patients the IBD subtype classification was estimated with a probability greater than 80% (Fig. 3D). Exploring the distribution of the posterior probabilities (Fig. 3D), patients are not equally distributed across the entire probability range. The sigmoidal distribution reflects higher certainty of the model predication where patients present with a pattern learnt during the construction step but prediction accuracy declines rapidly for patients exhibiting previously unseen patterns.

## Discussion

In this study we have mathematically modelled endoscopic and histological data to aid with classification of IBD diagnosis in paediatric patients. The resulting model demonstrates high accuracy in discriminating CD and UC patients and also provides an effective visualization of the complex overlap of these two disease subtypes.

Interpretation of the unsupervised models confirms uncertainty in discriminating CD and UC subtypes with overlapping and undefined clusters based only on disease location. We observed a limited separation of Crohn’s disease and ulcerative colitis patients, with UC presenting less variance than CD cases.

Based on the endoscopic and histological disease location the unsupervised models did not classify disease into distinct CD/UC subtypes, instead four distinct groups of patients were characterised by different colorectal involvement. The hierarchical clustering was not able to fit some individuals in those previously described groups. There are clear challenges in diagnostic categorisation based solely on disease location, however this model points to further subcategorization of disease, with significant overlap between UC and CD in groups i and ii. Whilst group iv is almost exclusively CD all colonic involvement has some overlap between disease types suggesting sub-classification of disease may be useful in distinguishing subtypes of CD or UC, potentially with impacts on management decisions. This theory has been raised previously through mathematical modelling of complex IBD data including serological and genetic markers^13, 29^. Regression analysis of CRP level at diagnosis with groups i-iv indicates a statistically significant increase in CRP in group iii, whilst the reason behind this are uncertain there is a need to identify patients with increased systemic inflammation in order to optimise treatment. Here we provide potential evidence of the need for further subcategorization of disease based on solely on clinical parameters used in standard practice.

It is well established that ileal inflammation is key to diagnosis of Crohn’s disease. Here we found that ileal inflammation (endoscopic or histological) is the only feature selected as important in all the models we constructed, providing evidence that ileal disease is the single most important factor for disease classification. Additionally, whilst colonic inflammation is important in paediatric UC, we find that it is also frequently present in CD with significant overlap between the 2 diseases.

There is significant interest in application of machine learning to clinical problems to aid with diagnosis, disease classification and personalising treatment. Nevertheless, the main focus of machine learning should not be to replace the human decision-making but to provide help in uncertain situations. There will always be an innate limitation of mathematical models to replicate the human intuition built with experience. However, some examples of machine learning applied to clinical data have been proved successful in situations to such as providing risk scoring systems^30^, imaging interpretation^31^, new patient stratification models^32^ and diagnostic tools^33^.

Our machine learning models have been utilised for solving a classification problem (CD vs UC) and additionally to observe data structure and complexity with a view to improvement of current classification. Through the application of machine learning to these data we confirmed the higher accuracy of histological over endoscopic data if used in isolation. We also demonstrated that both investigations are needed for an optimal classification, although the current Paris classification only accounts for endoscopic disease location.

Recently there has been interest in discrepancies between endoscopic and histological disease extent, with some calls to review the Paris classification of paediatric IBD to incorporate an additional histological score^4–6^. This model provides further evidence to suggest that there are significant differences between endoscopic and histological disease extent, with notable differences seen in Fig. 2C. Additionally the classification accuracy of the model of endoscopic disease alone is less than a combined model, further raising the need to discuss a modification to the Paris classification.

The potential clinical utility of machine learning models such as the one we have developed are significant, by placing these basic data into the model a clinician will get a disease probability score at this, the model is open to incorporating additional data coming from independent clinics, leading to increasing accuracy over time.

IBDU presents an ongoing challenge to clinicians. There is broad guidance on treatment but increasingly there is uncertainty with diagnosis and reclassification of disease at a later stage^20^. The model described here has been developed in an attempt to classify Crohn’s disease and Ulcerative Colitis at diagnosis, and not to reclassify IBDU based on disease location. Despite this, IBDU patients appear throughout the PCA/MDS plots and do not cluster, indicating a heterogeneous disease phenotype. We applied the model to 29 patients diagnosed with IBDU at initial endoscopy, 17 of these patients were assigned a probability of greater than 80% to either CD or UC based on their disease location. Posterior probabilities obtained from the classification of IBDU patients as either CD or UC, resulted in either high (p > 0.85, n = 14) or low (p < 0.65, n = 10) values, with few (n = 5) exceptions. This distribution suggests the presence of at least two subgroups within IBDU patients. The first, where the model assigns the CD/UC label with high confidence, might represent a subset of patients with a clinical presentation similar to those already observed and learnt in CD and UC cases. The second subgroup, labelled with low confidence, might instead reflect a distinct clinical presentation that does not fit in the current classification criteria. Support from ML modelling may be particularly attractive for IBDU cases.

The strengths of this study lie in the robust nature of data collection. Patients recruited to this study were diagnosed by 4 different clinicians from Southampton Children’s Hospital, therefore the pattern discovered by the model is not that of a single gastroenterologist. The supervised model combines different machine learning elements, but its relative simplicity makes it quick and easily interpretable. The feature selection step (RFE-CV) implicated the most informative GI locations for diagnosing IBD subtypes.

Through this model we report a diagnostic accuracy of 82.7% with an area under the ROC curve of 0.87, although for clinical application this would need to be increased to exceed 0.95. This may be possible with the addition of more patients or more data (e.g. blood data, granulomata). Comparing the metrics of the trained model with the performance over the validation set we conclude that: (1) the combined model performs better than individual histology or endoscopy models; (2) that both endoscopic and histological evidences are needed for an optimal classification of PIBD and (3) performance over the validation set is similar to that observed over the test set, confirming the absence of overfitting and good generalisation. Moreover, performance metrics seen in the validation set, suggest that classification of UC patients is much more complex than for CD patients, reflecting the uncertainty observed in clinics. In total, 94% of Crohn’s disease patients were successfully labelled as CD while only 65% of UCs were correctly labelled. In conclusion, the missing 17% percent in accuracy can be mostly attributed to a lower discriminability of patients affected by UC. Additionally, this work can be seen as a blueprint for improvement of IBD categorisation in the future, through modelling of additional data, such as variants from whole-exome sequencing, transcriptome profiles and microbiome signatures it may be possible to gain further, clinically relevant, disease groups^34^. In the future this may aid with treatment selection, prognostication and ongoing management.

This study employs a mathematical model of histological and endoscopic data within IBD; it provides a model with high diagnostic accuracy on unseen data (83.3%). We present 4 novel subgroups of disease identified by unsupervised machine learning based on colonic disease.

The purpose of this study was two-fold, to better understand disease aetiology, heterogeneity and classification and to understand the potential for machine learning to assist with disease classification. Through further work machine learning can aid clinicians to accurately subtype disease and personalise treatment. Additionally this may help with classification of IBDU. Whilst existing methods for diagnosis appear robust, the opportunity to improve and personalise therapy for patients through new and more accurate subtyping of disease is exciting and increasingly tangible.

## Electronic supplementary material

Dataset 1
